# A decade of the hostile environment and its impact on health

**DOI:** 10.1177/01410768221078327

**Published:** 2022-02-09

**Authors:** Ryan Essex, Ayesha Riaz, Seb Casalotti, Kitty Worthing, Rita Issa, James Skinner, Aliya Yule

**Affiliations:** 1 Institute for Lifecourse Development and School of Law and Criminology, The University of Greenwich, London, SE9 2UG, UK; 2 DocsNotCops, London, N1 6HT, UK; 3 University College London, London, WC1N 1EH, UK; 4 Medact, London, N1 6HT, UK; 5 Migrants Organise, London, W10 6TT, UK

In 2012, the former Home Secretary Theresa May introduced a set of policies, the intent of which were to ‘create a really hostile environment for illegal immigrants’^1^ (Note: While mechanisms existed to charge migrants prior to this, the practice did not become widespread until after 2012). These policies, now widely known as the hostile environment, refer to a range of measures that embed immigration control within a range of public and private services. Among these measures, the UK government requires landlords, employers, public servants, including police, teachers and healthcare workers to check people’s immigration status before they can offer housing, a job or healthcare. Those that fail to check people’s status can face fines or criminal sanctions signifying that immigration controls now permeate a range of essential services, with the effect of criminalising the day-to-day activities of undocumented migrants.

While the hostile environment has had sweeping force, its impact on health and the provision of healthcare has been particularly concerning as it undermines the founding principles of the National Health Service (NHS). Building on an asylum system which had become increasingly restrictive under both Labour and Conservative leaderships since the 1990s, and piggy-backing on the existing and pervasive rhetoric of ‘illegals’ and ‘healthcare tourists’, the then government seized the opportunity to reify restrictions to accessing healthcare through a raft of legal changes that were brought forward in 2014 and 2017. In 2017, upfront charging was introduced in hospitals for secondary care funded community services. This meant that unless an individual’s condition was considered urgent or immediately necessary, they were charged 150% of the estimated cost of their treatment upfront and treatment could be withheld if they did not pay. If treatment was urgent, an individual could be treated in the NHS and billed afterwards.^2^ NHS Trusts now employ dedicated teams of overseas managers who are responsible for upfront charging which in practice targets refused asylum seekers, undocumented migrants and others caught up by the dysfunctional immigration system, selling their data to bailiffs who go on to harass them.^3^ In addition to this, the government has also pushed several data-sharing arrangements within the NHS and externally with other public services, that enable patient data from the NHS to be used for immigration enforcement purposes.^4^ Today, if an individual has a debt of over £500 for two months or more, this is reported to Home Office by NHS trusts; this can impact on future visa applications and can be used to trace, detain and deport the individual(s) in question.

Perhaps unsurprisingly the hostile environment has caused destitution and ill-health. In practice, urgent and immediately necessary care is often wrongly delayed and withheld from vulnerable patients, often because of confusion related to what constitutes ‘urgent and immediately necessary treatment’. People are deterred from seeking treatment,^5^ with many fearful of potentially being detained and deported.^6^ Thousands more have been wrongly turned away from services.^7^ Much of this has been embodied in the recent ‘Justice for Simba Campaign’. Simba, a refused asylum seeker was receiving treatment for a blood clotting condition, but stopped attending treatment after he found out he would be charged (and that his bill might impact his future immigration applications), and after he was repeatedly phoned by the NHS Trust asking him for payment upfront before appointments. After stopping treatment, Simba suffered a stroke in 2019 and was billed £100,000 for the care he received under the NHS.^8^ For healthcare professionals, these policies raise a range of clinical and ethical issues, at the heart of these is the risk of being co-opted to act with policies that clearly undermine health and wellbeing, as a de-facto border guard. A survey found that numerous doctors had faced pressure from administrative staff in regard to patients’ need for care; up front charging also increased workload and took time away from patient care.^9^ This observation, that upfront charging is less efficient and even generates economic losses has been supported by other research,^10^ that suggest that the hostile environment is not about austerity, but rather about weaponising public services such as the NHS^11^ to appeal to some broader sense of xenophobia.^10^

The hostile environment has also impacted health outside of the NHS with its repercussions continuing to be felt today. For example, the response to the 2017 Grenfell fire cannot be understood without considering the wider implications of the hostile environment. Families of victims of this tragedy and survivors from Iran and Syria were initially prevented from receiving emergency visas to enter the UK.^12^ Furthermore, several survivors decided against seeking medical assistance or legal aid as they were concerned about their immigration status.^13^ Then there was Brexit. With the slogan of ‘taking back control of our borders’, the UK government has ended free movement for European migrants, paving the way for the Government’s ‘points based system’.^14^ At the time of writing, the UK government is exploring further possibilities which will marginalise and punish migrants, as it considers offshore processing, notwithstanding the disastrous impact this has had elsewhere in the world.^15^ This comes alongside the widely criticised use of ex-army barracks to accommodate asylum seekers and the more recent proposals to turn back boats of people attempting to cross the English Channel.^16^ This of course has all happened while the UK continues to come to terms with the COVID-19 pandemic, an issue which has disproportionality impacted those most marginalised and only further re-enforced the need for universal access to healthcare.

Despite this relatively pessimistic outlook, there is cause for hope; over the last decade these policies have been met with resistance not only from the healthcare community but also from those that have been directly impacted by these hostile and discriminatory policies. Building on the history of patient and worker resistance^17^ to the introduction of charges for healthcare, there has been concerted resistance to charging policies across the country, which has been best exemplified by the ‘Patients Not Passports’ campaign. This campaign has taken direct action, engaged in workplace education conducted research on the deleterious effects of the and charging regime. In 2019, the British Medical Association passed a motion at its annual conference to scrap the legislation that enabled NHS Trusts to charge those otherwise ineligible. More recently the Royal College of Paediatrics and Child Health has also come out and called for the wholesale scrapping of charging policies, adding to the calls already made by other major medical bodies to suspend them.^18^

A number of services exist to fill the gaps created by the hostile environment, including – for example – the Doctors of the World Clinic in Bethnal Green, and countless frontline migrant support organisations that are working to navigate extremely complex charging regulations and supporting people to access healthcare. A number of grassroots, migrant and healthcare worker-led networks also exist: Medact, Docs Not Cops, Migrants Organise, Doctors of The World, the Médecins Sans Frontières Take Action group, Hands Up For Health, among others. Guidance has been produced for clinicians attempting to navigate hostile environment regulations/legislation^19^ and assertively advocate for patients who are subject to these policies.^20^ Additionally, the ‘Vaccines For All’ campaign was established to counteract the effect of deterring people from accessing vaccines (for COVID-19) caused by the hostile environment. Doctors of The World has also set up a specific service to assist those who were wrongly charged for care related to COVID-19.

A decade on, what has changed? Quite a lot, but at the same time, very little. Arguably if anything, more recent policies and rhetoric suggest an increased brazenness on the part of the government and are indicative of a shift to, rather than a hostile environment, an abusive one. The last decade has witnessed multiple (and many completely avoidable) tragedies, such as the Grenfell Fire tragedy, the Windrush scandal and, most recently, the COVID-19 pandemic. The impact of these policies cannot be disentangled from the hostile environment. Sadly, on the same trajectory we should rightly be sceptical about the next decade. In saying this, attempts to introduce restrictive charging in healthcare are not new and historically in the UK and throughout Europe, healthcare workers have played a formative role in resisting these policies.^20^ Given the current course of the UK government, it seems that only through collective action will patient rights be protected and the hostile environment resisted. One can hope that after another decade, we are not having this conversation and that these policies are scrapped and replaced by ones that move the NHS toward the truly universal ideas ideals upon which it was founded.

## Declarations

### Guarantor

RE.

### Provenance

Not commissioned; peer-reviewed by Lara Miriam Ibrahim.

### ORCID iDs

Ryan Essex https://orcid.org/0000-0003-3497-3137

Seb Casalotti https://orcid.org/0000-0001-5463-9924
